# Evaluating agroclimatic constraints and yield gaps for winter oilseed rape (*Brassica napus* L.) – A case study

**DOI:** 10.1038/s41598-017-08164-x

**Published:** 2017-08-10

**Authors:** Zhi Zhang, Jianwei Lu, Rihuan Cong, Tao Ren, Xiaokun Li

**Affiliations:** 1 College of Resources and Environment, Huazhong Agricultural University, Wuhan, 430070 China; 2 Key Laboratory of Arable Land Conservation (Middle and Lower Reaches of Yangtze River), Ministry of Agriculture, Wuhan, 430070 China

## Abstract

Evaluating the effects of agroclimatic constraints on winter oilseed rape (WOSR) yield can facilitate the development of agricultural mitigation and adaptation strategies. In this study, we investigated the relationship between the WOSR yield and agroclimatic factors using the yield data collected from Agricultural Yearbook and field experimental sites, and the climate dataset from the meteorological stations in Hubei province, China. Five agroclimatic indicators during WOSR growth, such as ≥0 °C accumulated temperature (AT-0), overwintering days (OWD), precipitation (P), precipitation at an earlier stage (EP) and sunshine hours (S), were extracted from twelve agroclimatic indices. The attainable yield for the five yield-limiting factors ranged from 2638 kg ha^−1^ (EP) to 3089 kg ha^−1^ (AT-0). Farmers (*Y*_*farm*_) and local agronomists (*Y*_*exp*_) have achieved 63% and 86% of the attainable yield (*Y*_*att*_), respectively. The contribution of optimum fertilization to narrow the yield gap (*NY*_*exp*_) was 52% for the factor P, which was remarkably lower than the mean value (63%). Overall, the precipitation was the crucial yield-limiting agroclimatic factor, and restricted the effect of optimizing fertilization. The integrated data suggest that agricultural strategies of mitigation and adaptation to climatic variability based on different agroclimatic factors are essential for improving the crop yield.

## Introduction

Despite development in agricultural management and sustainable adaptation, agricultural production is still influenced by climatic, agronomic and/or socio-economic issues^1–3^. For climatic characteristics, radiation and temperature are yield-defining factors, rainfall and evapotranspiration are yield-limiting factors, and climatic conditions for pest/disease attacks are yield-reducing factors^4^. Oilseed rape, an oil and energy crop, is vulnerable to local climatic conditions because of its lengthy growth period and overwintering ability. China produces approximately 20% of world’s rapeseed^5^, and the Yangtze River Basin, which has a subtropical monsoon climate, is the major production region for winter oilseed rape (WOSR). Oilseed rape yields have been stagnant in several European countries since the mid-1980s, and one of the reasons is related to the crop’s growing environment^6^. Weather conditions have explained approximately 40% of the WOSR yield variability during specific growth phases in Germany^7^. In the case of China, WOSR encountered not only temporal yield stagnation from 2004 to 2014 but also spatial yield variability at provincial level^8,9^. Optimum fertilization has contributed greatly to WOSR yield^10,11^, but inadequate and excessive fertilization is common in Yangtze River Basin^12^. Identifying the agroclimatic constraints under climatic variability can help to explain the reasons for these yield issues.

The coincidence of variations in yield and climate was frequent for seed producing crops^1^. A series of agroclimatic indices can be used to analyze the interactions between crop growth and climate variability, such as the decrease in the rice yield with the increase in the minimum temperature at the International Rice Research Institute Farm^13^, the positive effects of a moderate decline in precipitation on wheat production^14^, and the influence of extreme temperatures on rice yield in southern China^15^. In addition, analytical methods involving multiple variables were adopted to identify site-specific yield-limiting factors^16–18^. Several studies have reported oilseed rape growth and yield responses to climatic parameters, and they have provided the biophysical basis of these factors^1,19–22^. Specifically, the temperature determines leaf area growth at early stage and the flowering period duration^23^ (Habekotté 1997), low temperatures prolong the post-flowering phase but increase radiation interception^1,24^, and limited water availability reduces the total dry matter production^7^.

To provide insight into the relationship between the spatial yield variability and the regional climatic characteristics, scientific observations about the agroclimatic constraints on WOSR yield are essential. The objectives of this paper were to (i) develop agroclimatic indices representing the effects on rainfed WOSR growth and extract the dominant agroclimatic factors, (ii) identify the regional agroclimatic limiting factors and quantify yield losses, and (iii) estimate yield gaps attributed to the limiting factors and evaluate yield gap mitigation.

## Results

### Selection of the minimum agroclimatic dataset

**Figure 1:**
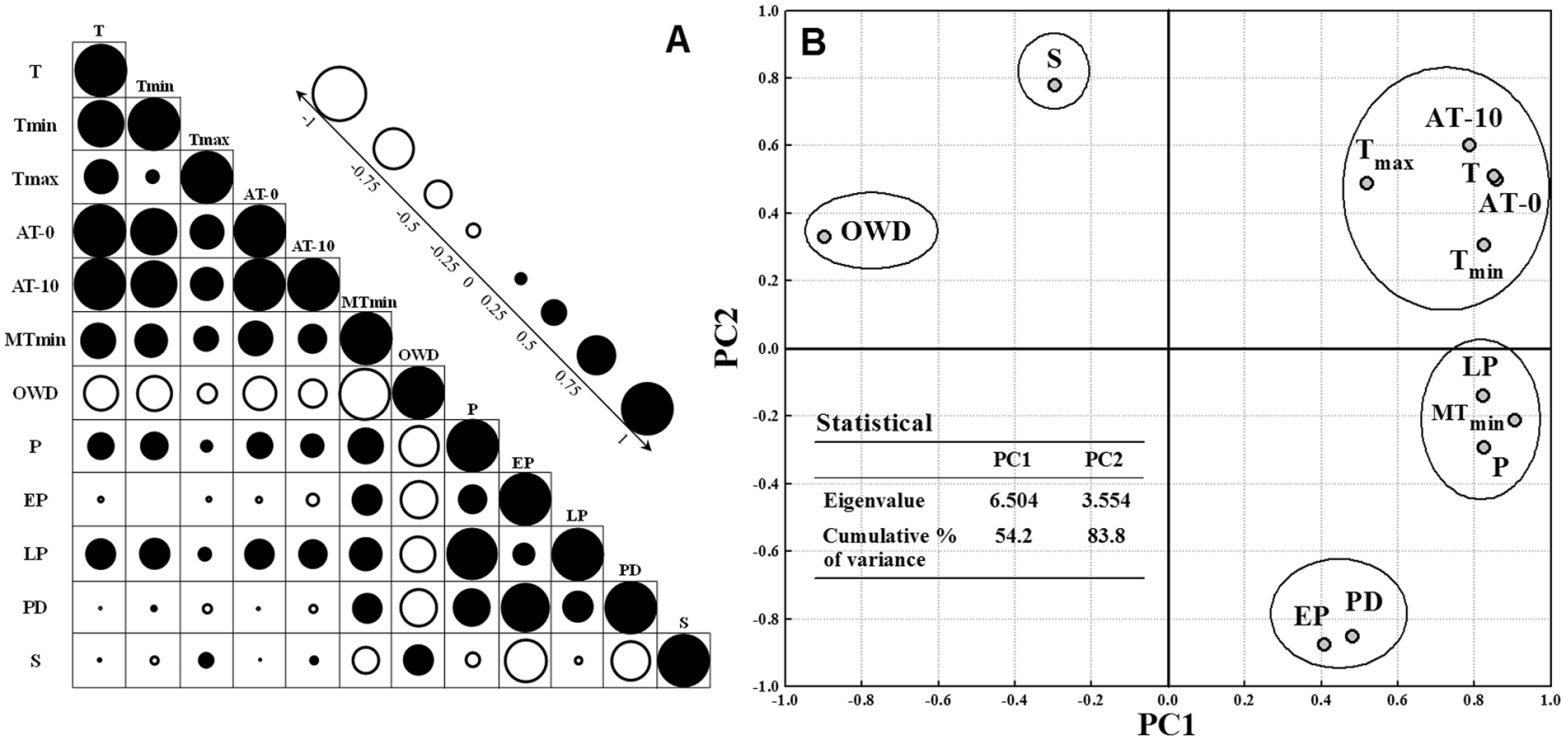
Correlation coefficient matrix (**A**) of the meteorological factors and loading distribution (**B**) in an extracted principal component analysis. T mean temperature, T_min_ mean daily minimum temperature, T_max_ mean daily maximum temperature, AT-0 mean ≥0 °C accumulated temperature, AT-10 mean ≥10 °C accumulated temperature, MT_min_ mean minimum mean monthly temperature, OWD mean overwintering days, P mean precipitation, EP mean precipitation at an earlier stage, LP mean precipitation at a later stage, PD mean precipitation days, S mean sunshine hours.

The correlation coefficient matrix for the twelve agroclimatic indices is shown in Fig. 1A. Clearly, there were some high positive and negative correlations present. Two principal components (PC) were extracted, and the cumulative variance was 83.8% (Fig. 1B). PC1 and PC2 described 54.2% and 29.6% of the total variance, respectively. There were five parts in the factor loading distribution in the first, second and fourth quadrants (Fig. 1B). In the first quadrant, a ≥ 0 °C accumulated temperature (AT-0) was chosen as the high loading factor for PC1, which has the most significant correlation among the indices concerning temperature. In the second quadrant, both the overwintering days (OWD) and sunshine hours (S) were chosen as the high loading factor for PC1 and PC2, respectively. In the fourth quadrant, precipitation (P) was chosen from precipitation at a later stage (LP) and minimum mean monthly temperature (MT_min_) for its higher correlation coefficient and acceptability. Additionally, precipitation at an earlier stage (EP) was chosen as a high loading factor for PC2. Finally, five agroclimatic indicators (AT-0, OWD, P, EP and S) were chosen as the dominant factors.

### Boundary line analysis of the dominant agroclimatic factors

**Figure 2:**
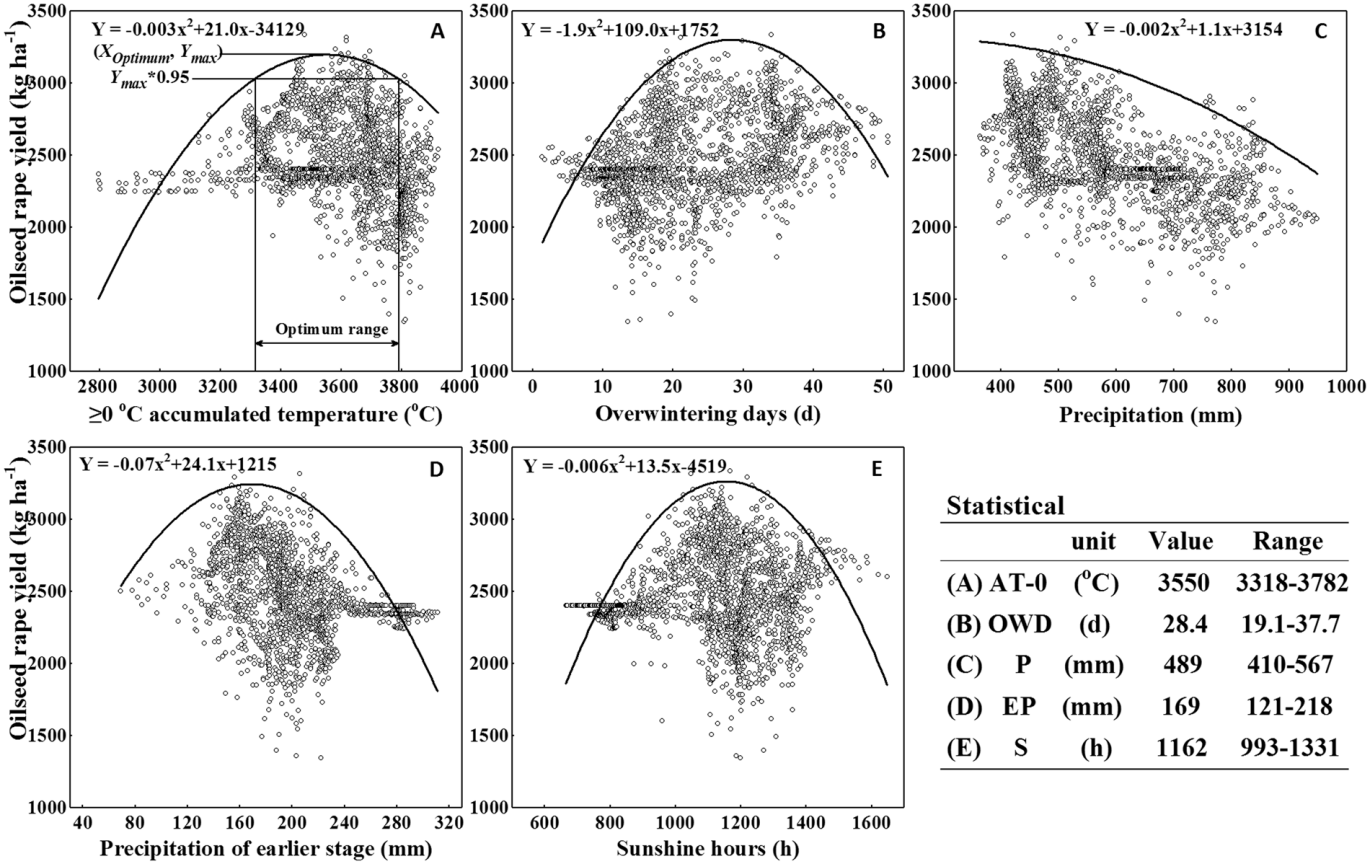
Relationship between the winter oilseed rape yield and the dominant agroclimatic factors. (**A**) AT-0, ≥0 °C accumulated temperature; (**B**) OWD, overwintering days; (**C**) P, precipitation; (**D**) EP, precipitation at an earlier stage; (**E**) S, sunshine hours. The lines represent the boundary lines (n = 2144).

Boundary regression lines were determined by the upper boundary points for the five factors (Fig. 2). For AT-0, OWD, EP and S, the winter oilseed rape (WOSR) yields increased until the maximum value, followed by a decrease. For factor P, the WOSR yield declined with increasing precipitation. The optimum values and ranges of the dominant agroclimatic factors could be calculated on the basis of the boundary lines. The optimum values of AT-0, OWD, P, EP and S were 3550 °C, 28.4 d, 489 mm, 169 mm and 1162 h, respectively. Considering the threshold of *Y*_max_*0.95^16^, the optimum ranges of all five factors are shown in Fig. 2. It is worth noting that the optimum precipitation range started at 410 mm, at which the actual yield was the maximum. The actual yield was significantly lower than the predicted yield at a precipitation quantity of less than at 410 mm, below which crop drought may occur.

**Figure 3:**
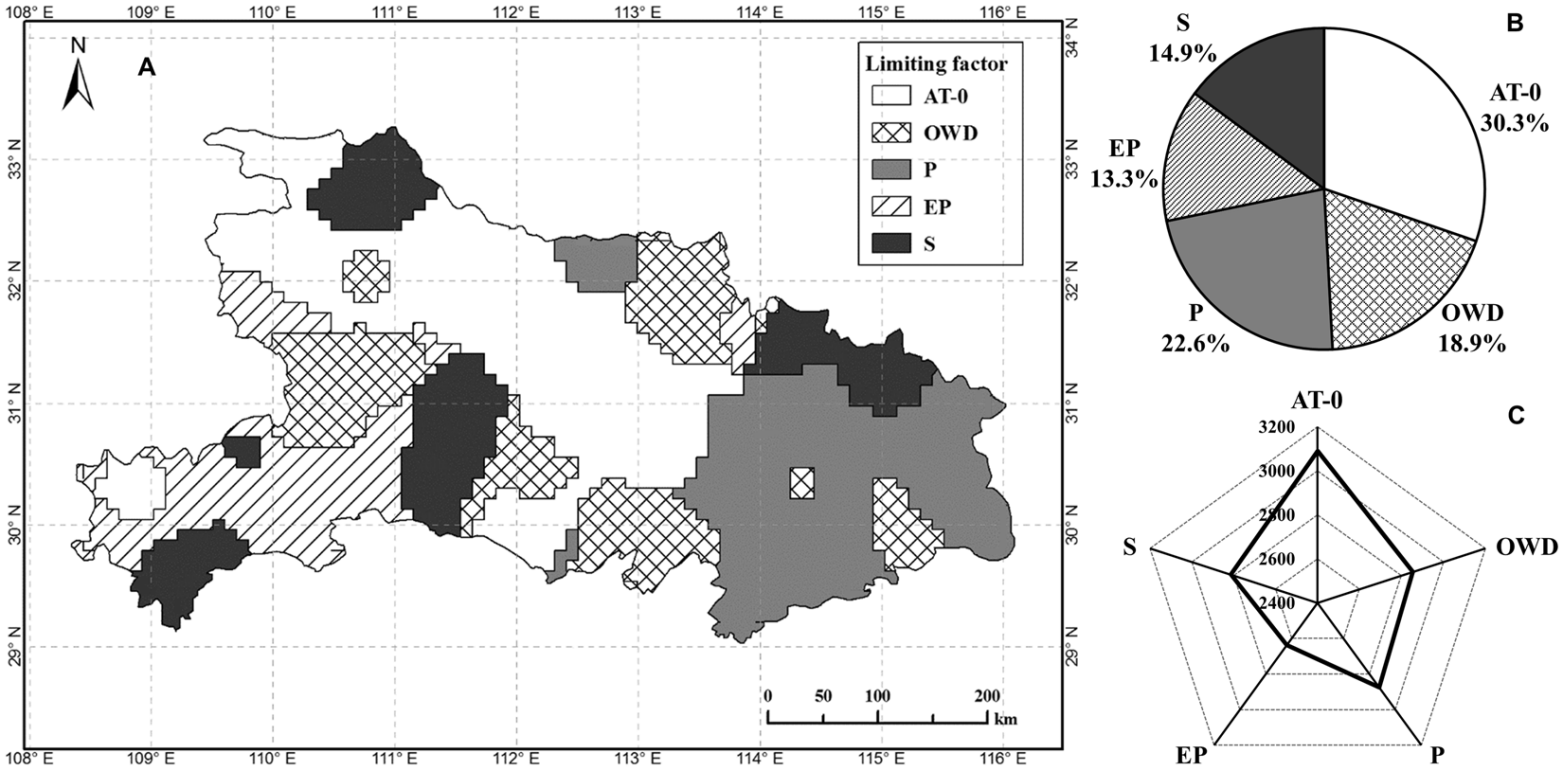
Spatial distribution (**A**) and proportion (**B**) of yield-limiting agroclimatic factors, and the attainable yield as predicted by the multivariate model. AT-0 mean ≥0 °C accumulated temperature, OWD mean overwintering days, P mean precipitation, EP mean precipitation at an earlier stage, S mean sunshine hours. Figure was created by ArcGIS Desktop (Version 9.3, URL: http://www.esri.com) [Software].

The yield-limiting agroclimatic factors were identified using the multivariate equation (equation 2) for each grid point (Fig. 3). The most widespread of the limiting factor across the region was AT-0, which accounted for 30.3% of the grid points and mainly distributed in the north-central of the region. Secondly, the limiting factor P accounted for 22.6% of the grid points, and distributed in the southeast. The spatial distribution of OWD (18.9%), S (14.9%) and EP (13.3%) were relatively random as the limiting factor.

As for the agroclimatic constraints, the average value of the attainable yield (*Y*_*att*_) was 2,854 kg ha^−1^ in the region (Fig. 3C). For the five different agroclimatic factors, EP had highest impact on the *Y*_*att*_ (2638 kg ha^−1^, which refers to the attainable yield under the limiting factor of EP), followed by OWD, P and S (approximately 2,800 kg ha^−1^), with the lowest effect for AT-0 (3,089 kg ha^−1^). These results indicate that the limiting factor of the temperature accumulation appeared to affect the yield within a large area, but led to a small yield loss, while precipitation at an earlier stage (generally from September to November) appeared to affect the yield within a small area, but resulted in a large yield loss.

### Winter oilseed rape yield

**Figure 4:**
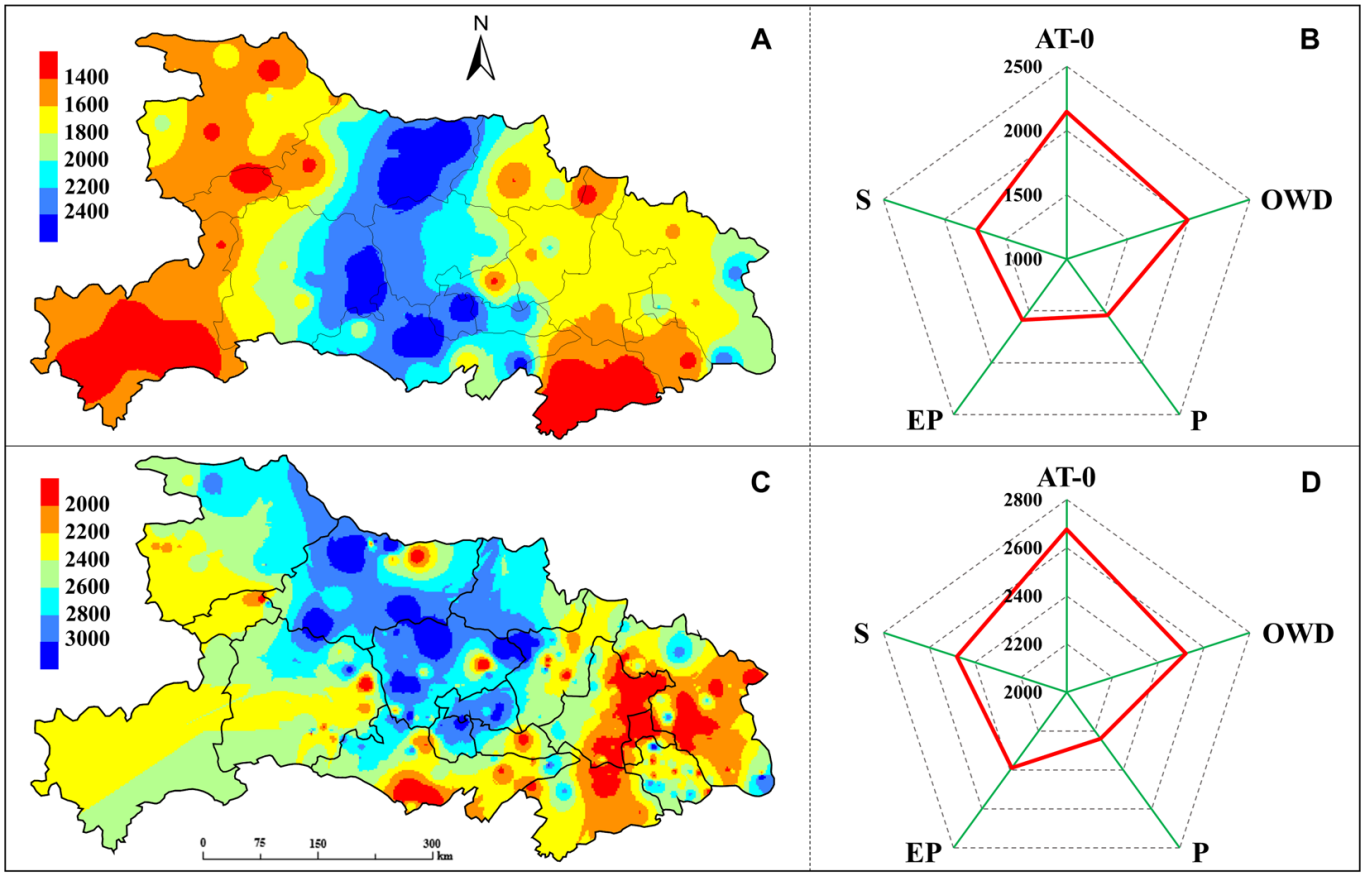
Spatial distribution of the actual farmers’ yield (**A**) and the experimental yield (**C**), and the values for different yield-limiting agroclimatic factors (**B** and **D**). AT-0 mean ≥0 °C accumulated temperature, OWD mean overwintering days, P mean precipitation, EP mean precipitation at an earlier stage, S mean sunshine hours. Figure was created by ArcGIS Desktop (Version 9.3, URL: http://www.esri.com) [Software].

The spatial distribution of the actual farmers’ yield (*Y*_*farm*_, averaged 1800 kg ha^−1^) and the experimental yield (*Y*_*exp*_, averaged 2461 kg ha^−1^) are shown in Fig. 4A and C, respectively. The high-value *Y*_*farm*_ (>2200 kg ha^−1^) was mainly distributed in the central of the region, and the low-value *Y*_*farm*_ (<1600 kg ha^−1^) was in the southeast and the west. Similarly, the high-value *Y*_*exp*_ (>2800 kg ha^−1^) was mainly distributed in the north-central, and the low-value *Y*_*exp*_ (<2200 kg ha^−1^) was in the southeast.

The two yield benchmarks for different yield-limiting agroclimatic factors were obtained by overlaying their spatial distribution maps. For the five factors, the *Y*_*farm*_ ranged from 1,540 kg ha^−1^ to 2,149 kg ha^−1^, and the *Y*_*exp*_ ranged from 2,638 kg ha^−1^ to 3,089 kg ha^−1^. Coincidentally, the highest values of *Y*_*farm*_ and *Y*_*exp*_ were both observed in the limiting factor of AT-0, and their lowest values were both observed in the limiting factor of P (Fig. 4B and D). By comparing the two yields, optimum fertilization was found to improve the farmers’ yield by 25~51%, with the largest value found in the limiting factor of EP.

### Winter oilseed rape yield gap

**Figure 5:**
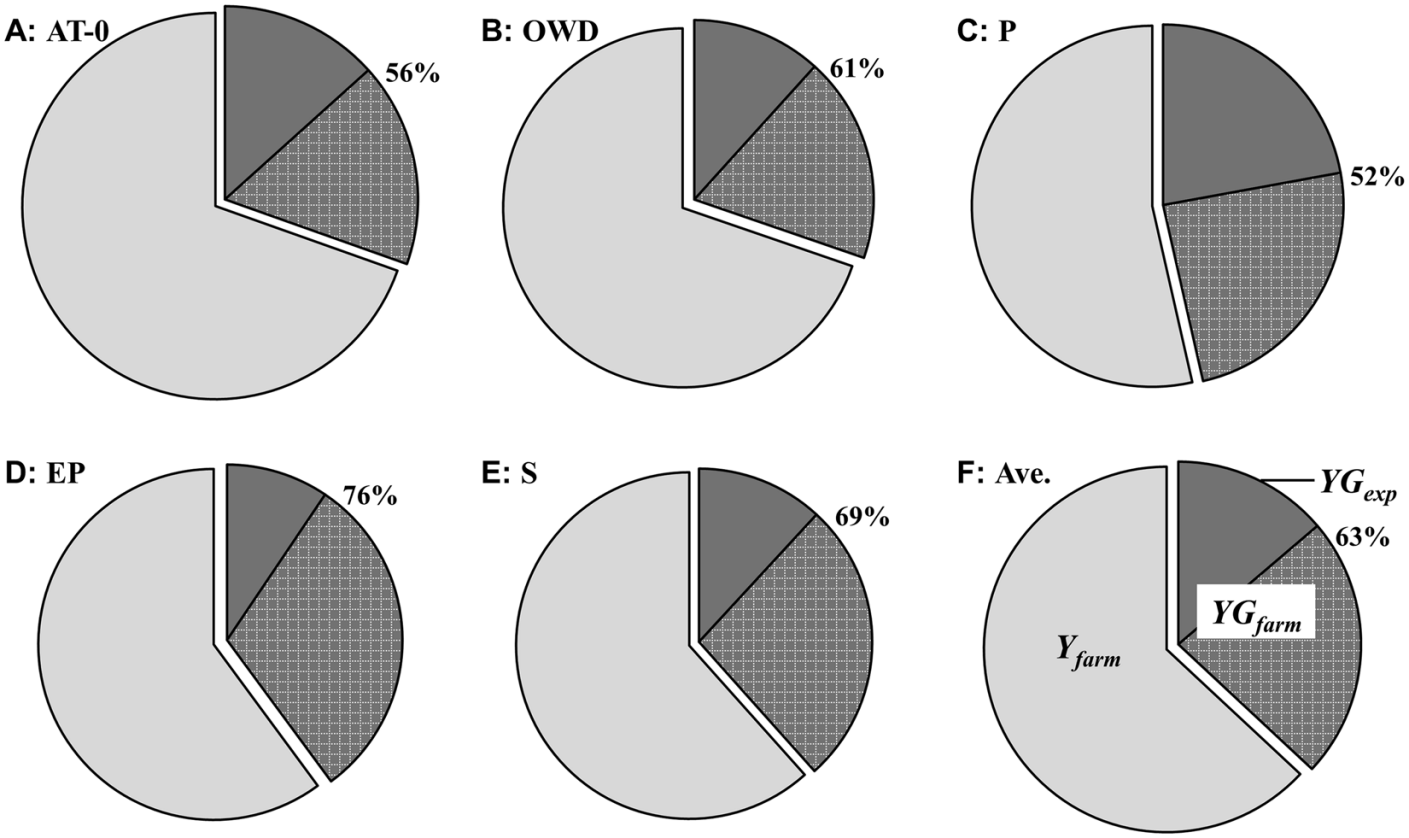
Yield gaps for different yield-limiting agroclimatic factors. (**A**) AT-0, ≥0 °C accumulated temperature; (**B**) OWD, overwintering days; (**C**) P, precipitation; (**D**) EP, precipitation at an earlier stage; (**E**) S, sunshine hours; (**F**) Ave., whole region. The size of pie chart represents the *Y*_*att*_, the pale gray part on the left represents the *Y*_*farm*_, the two dark gray parts on the right represent the *YG*_*farm*_, and the *YG*_*exp*_, and the percentage beside the pie chart represents the *NYG*_*exp*_.

The yield gaps of WOSR are presented in Fig. 5. The *YG*_*farm*_ (which was the difference between *Y*_*att*_ and *Y*_*farm*_) averaged 1,054 kg ha^−1^, which means the farmers achieved 63% of the *Y*_*att*_. Among the five limiting factors, the factor P had the highest value of *YG*_*farm*_ (1335 kg ha^−1^), while the factor AT-0 exhibited the lowest value (864 kg ha^−1^). For the *YG*_*exp*_ (which was the difference between *Y*_*att*_ and *Y*_*exp*_), the average value was 393 kg ha^−1^, which means the local agronomists achieved 86% of the *Y*_*att*_. In the different agroclimatic limiting regions, the factor P also had the maximum value of *YG*_*exp*_ (636 kg ha^−1^), and the EP had the lowest value (249 kg ha^−1^).

To assess the contribution of optimum fertilization to narrowing the yield gap, *NY*_*exp*_ was calculated using equation (5) and the results are shown in Fig. 5. The *NY*_*exp*_ averaged 63%, and ranged from 52% for the factor P to 76% for the factor EP. The results indicate that precipitation was the most significant factor in the yield-limiting agroclimatic factors. The effect of the limiting factor of EP on yield was weaken under the optimum fertilization.

## Discussion

The boundary line approach was appropriate for the analysis of agroclimatic data in the subtropical monsoon region (Fig. 2), which is consistent with the proposal by Wairegi *et al*.^25^ for a single agro-ecological zone^25^. Our findings showed that precipitation was the most important agroclimatic constraint, and the optimum range for seasonal WOSR was from 410 to 567 mm (Fig. 2). After 567 mm, an increase of 10 mm in precipitation corresponded to a decrease of 13 kg ha^−1^ in the attainable yield (data not presented), which was similar to that of winter wheat during the late growth phase in Finland^19^. The high correlation between precipitation and precipitation during the later stage (Fig. 1A) suggested that a yield reduction resulted from waterlogging from flowering to harvest^21,26^. In this case, irrigation was generally not needed, but drainage was indeed necessary for WOSR, which is consistent with the perceptions of local farmers. Post-anthesis growth is important for the growth of pods and seed filling^27^, and waterlogging could reduce yields by restricting the seed number or weight as reported in wheat^28^. In contrast, the precipitation during the early stage should receive due attention because early drought has been considered as a key limiting factor in crop production^29^. Water deficit could decrease the germination rate and prolong germination time, leading to the reduction of root mass and leaf area in vegetative growth^30^.

Farmers usually changed the sowing time or selected optimum cultivars to adapt to the variable temperature. Elevated temperatures advanced plant maturity and interfered with seed filling, in a decrease of 10% in the WOSR yield in some European countries with an increase of 3 °C in the average temperature during seed filling^1^. The low temperature affected seed germination, leaf emergence and generative development^27^. Vernalization was required for the vegetative stage of winter crops during the winter, and a warming period in the spring maintained the regrowth^20,27^. In this study, overwintering generally started in the middle of December and ended in February, and the mean temperature in this period was about 5 °C (data not shown).

The number of sunshine hours appeared to affect the WOSR yield to a slight extent (Fig. 3C), but it supported photosynthetic activity. A high negative correlation was shown between the sunshine hours and precipitation at the early stage (Fig. 1A), which suggested that sunlight shortage during the vegetative stage interfered with leaf photosynthesis. During the analysis of yield-limiting factors, the interactions between the agroclimatic factors and the diseases, pests and weeds caused by climate variability cannot be ignored. For example, the interactions between rainfall and weed management in cassava production^31^, and the pests and soil-related factors in banana production^25^ show their importance. The leaf number and dry matter accumulation were reduced by the low temperature and overcast weather that is common during the winter in China, and root growth was hindered by early water shortage and cool temperatures during WOSR production.

The yield gap concept in this paper was different from that of Lobell *et al*.^32^, i.e., the difference between average and potential yield, but similar to that of Wairegi *et al*.^25^ and Wang *et al*.^25,33^, i.e., the gap between attainable yield and minimum predicted yield in the given region. In this paper, we did not evaluate the potential yield of WOSR^4^, but the attainable yield instead. The highest reported yield of WOSR in the region was 4,829 kg ha^−1^ ^34^, implying the great potential for yield improvement. The total yield gap averaged 1,054 kg ha^−1^, and the experimental yield narrowed the gap by 63%, indicating that more efforts are needed to improve management practices. Optimum fertilization could save a significant yield loss in the Yangtze River Basin of China^10,11^, but adjusting management practices according to different weather conditions is more important for narrowing the yield gap.

Although the agroclimatic data and yield data were combined by the “site-area-point” method, the large number and extensive distribution of meteorological stations and experimental sites may result in uncertainties in the results. The selection of agroclimatic indices and the dominant factors were sometimes subjective, but they were consistent with expectation and experience. In future studies, agroclimatic indices can be more detailed with every growth period. In this study, we did not consider the influence of regional orography since WOSR was generally planted at a low altitude in Hubei province. We believe that these results are consistent with those of other similar climatological regions, and the analytical method is applicable to other climate zones.

## Conclusions

This paper has identified the agroclimatic limiting factors and quantified the agroclimatic-induced yield gap for WOSR in a subtropical monsoon climate. The yield variability was interpreted by the five dominant factors of ≥0 °C accumulated temperature, overwintering days, precipitation, precipitation at an earlier stage and sunshine hours, which were found to be the major limiting factors across the region. Although the accumulation temperature affected the WOSR yield over a wide area, the precipitation appeared to be the most important agroclimatic constraint in the region. Optimum fertilization effectively narrowed the actual yield gap, especially under the limit of precipitation at an earlier stage, but its efficiency was restricted significantly by the precipitation constraint. The agroclimatic constraints and yield gaps presented in this study provided a basis for the development of mitigation and adaptation measures to respond to climatic variability in combination with agricultural strategies.

## Methods

### Study area

**Figure 6:**
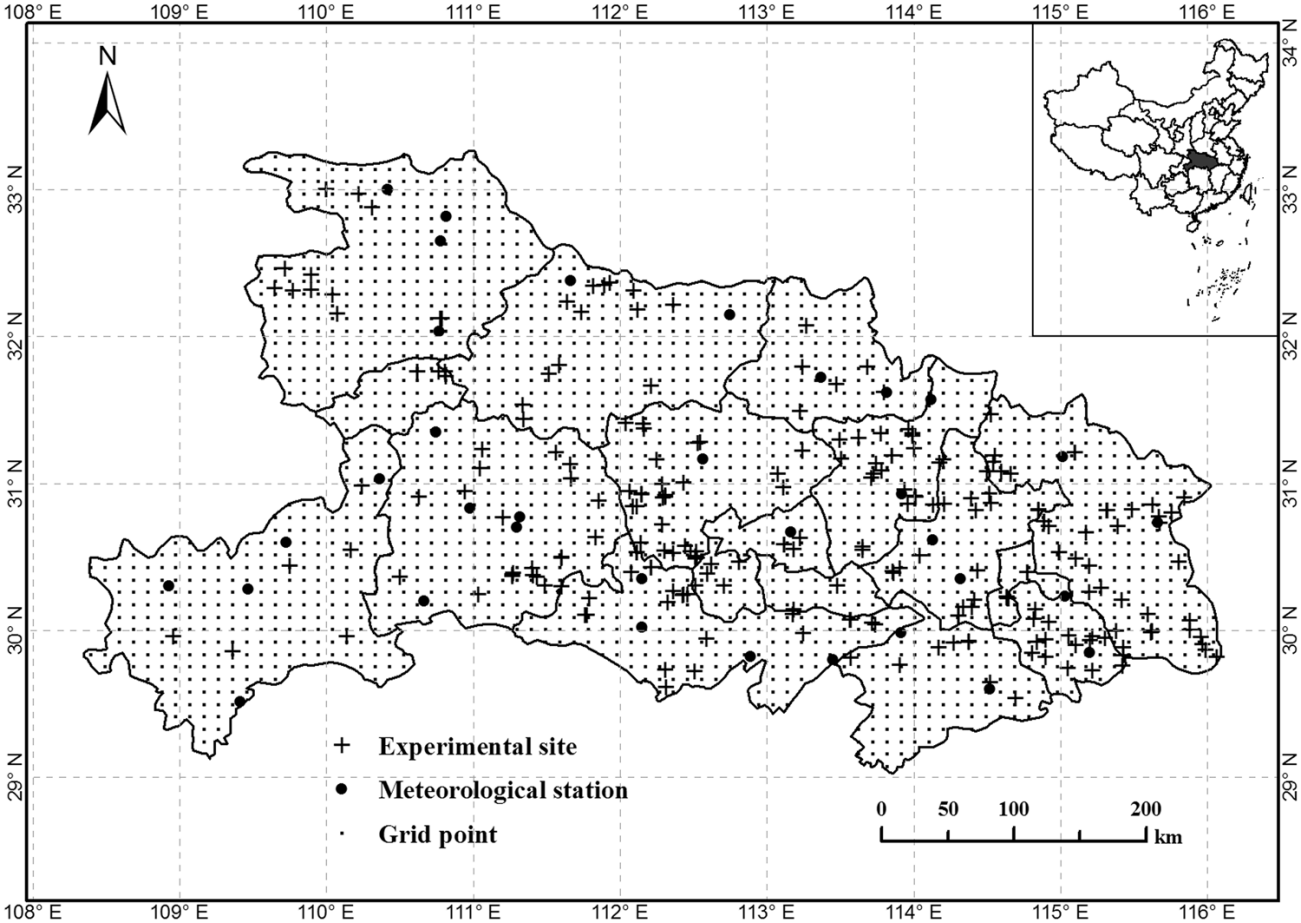
Distribution of meteorological stations, experimental sites and grid points for winter oilseed rape in Hubei province. Figure was created by ArcGIS Desktop (Version 9.3, URL: http://www.esri.com) [Software].

This study was conducted in Hubei province (Fig. 6) (29°05′–33°20′N, 108°21′–116°07′E), which is the largest winter oilseed rape (WOSR)-producing province of the Yangtze River Basin, China. The planting area (11.4 × 10^5^ hm^2^) and production (2.3 × 10^6^ t) accounts for 16% of China’s national WOSR production^8^. This province has a subtropical monsoon climate, with an average annual temperature of 16.7 °C and precipitation of 1313 mm. The average temperature and total precipitation during the WOSR growing season (generally from September to May of the following year) were 13.1 °C and 596 mm, respectively. WOSR was generally rotated with rice under a double cropping system.

### Data source

Daily climate variables (e.g., the average air temperature, maximum and minimum air temperatures, precipitation, and sunshine hours) were collected from 31 meteorological stations (Fig. 6) in Hubei province during the 2005–2014 period (http://data.cma.cn/).

The actual farmers’ yield (*Y*_*farm*_) data from 2005 to 2009 were collected from the Agricultural Yearbook of Hubei province^35^.

The experimental yield database were obtained from 2005 to 2009 from 245 field fertilization experiments conducted by the local agronomists. The yield data of optimum fertilization treatment was chosen to represent the experimental yield (*Y*_*exp*_). To compare with the farmers’ management, the fertilization practice, including fertilizer rate, the ratio of NPK, and nitrogen application, was optimized for the experimental condition. The variety, sowing date, density and other management practices were all similar to those of the local farmers’ fields. The growth period was approximately 220 days, generally from 10 September to 15 May the following year. To ensure the data quality, outliers with the harvest index >0.5 or <0.2 were excluded.

### Data analysis

#### Agroclimatic indices

Considering the physiological characteristics and the primary meteorological challenges to WOSR, twelve agroclimatic indicators for each meteorological station were selected in Hubei province (Table 1). The indicators covered the entire WOSR growing period, including germination, seedling formation, stem elongation, flowering, podding and maturation stages. The temperature, precipitation, and sunshine hours were calculated for the average of the whole growing cycle. The minimum mean monthly temperature was the average temperature of the coldest month (which generally occurred in January). The overwintering days were calculated by using the five-day sliding average method to determine the starting and ending time of overwintering period, with a temperature lower than 5 °C and a minimum temperature lower than 0 °C for five consecutive days defined as the starting day, and when the temperature conditions were not met, as the ending day. Precipitation at the earlier stage and later stage was calculated from sowing to the beginning of overwintering and from the beginning of flowering to the harvest, respectively.

**Table 1:** Description of the twelve agroclimatic indices for winter oilseed rape.

Name	Unit	Abbreviation	Mean	Min	Max
Temperature	°C	T	13.1	10.1	14.4
Daily minimum temperature	°C	T_min_	10.1	7.0	13.6
Daily maximum temperature	°C	T_max_	17.7	13.9	19.7
≥0 °C accumulated temperature	°C	AT-0	3595	2770	3923
≥10 °C accumulated temperature	°C	AT-10	3066	2220	3416
Minimum mean monthly temperature	°C	MT_min_	3.3	0.9	5.9
Overwintering days	d	OWD	23	1	51
Precipitation	mm	P	596	363	951
Precipitation at an earlier stage	mm	EP	203	69	311
Precipitation at a later stage	mm	LP	310	174	539
Precipitation days	d	PD	88	65	122
Sunshine hours	h	S	1133	663	1656

#### Spatial database

**Figure 7:**
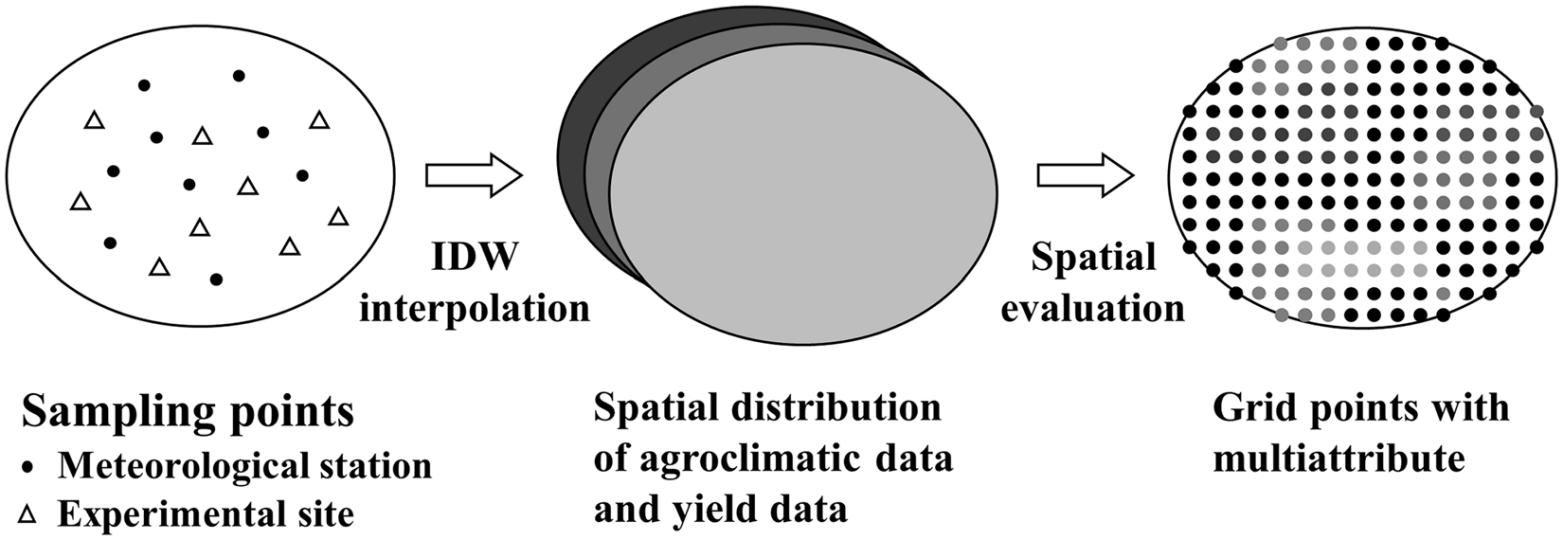
Construction process for the spatial vector database. The color of the circle represent the value of the indicator.

Spatial interpolation, including agroclimatic indicators and yield benchmarks, was performed by the inverse distance weighted (IDW) method using ArcGIS (version 9.3). The ‘Extract values to points’ tool (in ArcGIS) was used to convert raster data to vector data. The vector data were the uniform distribution points in Hubei province, which were divided into grid points (10 km × 10 km resolution) (Fig. 6). Grid cells of 50 km × 50 km or 0.5° × 0.5° resolution were extracted at a national scale^36,37^. A spatial vector database consisting of 2,144 grid points, including 12 agroclimatic indices and WOSR yield indices, was presented in the flow chart (Fig. 7). Therefore, the grid points (the combined meteorological data and yield data) were used in the following analysis.

#### Principal component analysis

In order to select the dominant factors from the twelve agroclimatic indices, principal component analysis (PCA) was adopted. The PCA was used to minimize the dimensionality of indicators and identify new and important underlying variables. Principal components (PC) with eigenvalues ≥1 and variation ≥5% were retained^38^. The dominant agroclimatic factors were selected by considering their higher factor loading in PCs as the best representative of the system. In addition, the correlation matrix and flexible norms were used on an auxiliary basis^39^ for the dominant factor selection.

#### Boundary line analysis

For each dataset related to the dominant agroclimatic factor (x-axis) and *Y*_*exp*_ (y-axis), upper boundary points (i.e. maximum value on each x-interval which was divided at ten intervals) were estimated from scatter plots using the boundary line development system (BOLIDES) established by Schnug *et al*.^16^. The maximum yields showed an increasing tendency first and then a decrease. Hence, the quadratic model was fitted through the upper boundary points as follows:

1 $$Y={\rm{a}}{x}^{2}+{\rm{b}}x+{\rm{c}}$$

where *x* is the independent variable; a, b and c are the constants. Each boundary line function was the maximum attainable yield (*Y*_*Xi*_) for each agroclimatic factor (*i* = 1, 2,…, n) in each grid point. For each grid point, the responses were assumed according to von Liebig’s law of the minimum^40^, and the minimum attainable yield (*Y*_*att*_, the attainable yield under the limiting factors) can be described by the multivariate model as follows:

2 $${Y}_{att}=\,{\rm{MIN}}({Y}_{X1},{Y}_{X2},\ldots ,{Y}_{Xn})$$

The limiting factor was identified as *Y*_*Xi*_ (*i* = 1, 2,…, n), corresponding to *Y*_*att*_.

#### Yield gap

To evaluate the yield gaps by comparison with *Y*_*att*_, two yield gaps based on different yield benchmarks were defined: *YG*_*farm*_ and *YG*_*exp*_, which were calculated using equation (3) and (4), respectively. Then, the contribution of optimum fertilization to narrow the yield gap (*NY*_*exp*_) was calculated using equation (5).

3 $$Y{G}_{farm}={Y}_{att}\mbox{--}{Y}_{farm}$$

4 $$Y{G}_{exp}={Y}_{att}\mbox{--}{Y}_{exp}$$

5 $$N{Y}_{exp}=({Y}_{exp}-{Y}_{farm})/Y{G}_{farm}$$
