# Publisher Correction: Evaluating agroclimatic constraints and yield gaps for winter oilseed rape (*Brassica napus* L.) – A case study

**DOI:** 10.1038/s41598-018-20636-2

**Published:** 2018-02-06

**Authors:** Zhi Zhang, Jianwei Lu, Rihuan Cong, Tao Ren, Xiaokun Li

**Affiliations:** 1 College of Resources and Environment, Huazhong Agricultural University, Wuhan, 430070 China; 2 Key Laboratory of Arable Land Conservation (Middle and Lower Reaches of Yangtze River), Ministry of Agriculture, Wuhan, 430070 China

Correction to: *Scientific Reports* 10.1038/s41598-017-08164-x, published online 10 August 2017

The original version of this Article incorrectly included Zhi Zhang as a corresponding author. For correspondence and requests for materials, please contact Rihuan Cong (congrh@mail.hzau.edu.cn). This error has now been corrected in the PDF and HTML versions of this Article.

